# PEGylated Chitosan Nanoparticles Encapsulating Ascorbic Acid and Oxaliplatin Exhibit Dramatic Apoptotic Effects against Breast Cancer Cells

**DOI:** 10.3390/pharmaceutics14020407

**Published:** 2022-02-13

**Authors:** Sherif Ashraf Fahmy, Asmaa Ramzy, Asmaa A. Mandour, Soad Nasr, Anwar Abdelnaser, Udo Bakowsky, Hassan Mohamed El-Said Azzazy

**Affiliations:** 1 Department of Chemistry, School of Sciences & Engineering, The American University in Cairo, AUC Avenue, P.O. Box 74, New Cairo 11835, Egypt; sheriffahmy@aucegypt.edu (S.A.F.); asmaaramzy95@aucegypt.edu (A.R.); 2 Pharmaceutical Chemistry Department, Faculty of Pharmacy, Future University in Egypt, Cairo 11835, Egypt; asmaa.abdelkereim@fue.edu.eg; 3 Institute of Global Health and Human Ecology, School of Sciences & Engineering, The American University in Cairo, AUC Avenue, P.O. Box 74, New Cairo 11835, Egypt; soad.nasr@aucegypt.edu (S.N.); anwar.abdelnaser@aucegypt.edu (A.A.); 4 Department of Pharmaceutics and Biopharmaceutics, University of Marburg, Robert-Koch-Str. 4, 35037 Marburg, Germany

**Keywords:** chitosan nanoparticles, PEG, oxaliplatin, ascorbic acid (vitamin C), ionic gelation method, breast cancer

## Abstract

This study aims to design a pH-responsive dual-loaded nanosystem based on PEGylated chitosan nanoparticles loaded with ascorbic acid (AA) and oxaliplatin (OX) for the effective treatment of breast cancer. In this regard, non-PEGylated and PEGylated chitosan nanoparticles (CS NPs) loaded with either ascorbic acid (AA), oxaliplatin (OX), or dual-loaded with AA-OX were fabricated using the ionotropic gelation method. The hydrodynamic diameters of the fabricated AA/CS NPs, OX/CS NPs, and AA-OX/CS NPs were 157.20 ± 2.40, 188.10 ± 9.70, and 261.10 ± 9.19 nm, respectively. While the hydrodynamic diameters of the designed AA/PEG-CS NPs, OX/PEG-CS NPs, and AA-OX/PEG-CS NPs were 152.20 ± 2.40, 156.60 ± 4.82, and 176.00 ± 4.21 nm, respectively. The ζ-potential of the prepared nanoparticles demonstrated high positive surface charges of +22.02 ± 1.50, +22.58 ± 1.85 and +40.4 ± 2.71 mV for AA/CS NPs, OX/CS NPs, and AA-OX/CS NPs, respectively. The ζ-potential of the PEGylated CS NPs was reduced owing to the shielding of the positive charges by the PEG chains. Additionally, all the prepared nanoparticles exhibited high entrapment efficiencies (EE%) and spherical-shaped morphology. The chemical features of the prepared nanoparticles were investigated using Fourier transform infrared (FTIR) spectroscopy. Release studies showed the capability of the prepared non-PEGylated and PEGylated chitosan NPs to release their cargo in the acidic environment of cancer tissue (pH 5.5). Furthermore, the AA/CS NPs, AA/PEG-CS NPs, OX/CS NPs, OX/PEG-CS NPs, AA-OX/CS NPs and AA-OX/PEG-CS NPs exhibited remarkable cytotoxic activities against breast adenocarcinoma (MCF-7) cells with IC_50_ values of 44.87 ± 11.49, 23.3 ± 3.73, 23.88 ± 6.29, 17.98 ± 3.99, 18.69 ± 2.22, and 7.5 ± 0.69 µg/mL, respectively; as compared to free AA and OX (IC_50_ of 150.80 ± 26.50 and 147.70 ± 63.91 µg/mL, respectively). Additionally, treatment of MCF-7 cells with IC_50_ concentrations of AA, AA/CS NPs, AA/PEG-CS NPs, OX, OX/CS NPs, OX/PEG-CS NPs, AA-OX/CS NPs or AA-OX/PEG-CS NPs increased the percentages of early apoptotic cells to 5.28%, 9.53%, 11.20%, 5.27%, 13.80%, 8.43%, 2.32%, and 10.10%, respectively, and increased the percentages of late apoptotic cells to 0.98%, 0.37%, 2.41%, 2.06%, 0.97%, 9.66%, 56%, and 81.50%, respectively. These results clearly indicate that PEGylation enhances the apoptotic effect of AA and OX alone, in addition to potentiating the apoptotic effect of AA and OX when combined on MCF-7 cells. In conclusion, PEGylated chitosan nanoparticles encapsulating AA, OX, or AA and OX represent an effective formula for induction of apoptosis in MCF-7 cells.

## 1. Introduction

Breast cancer is the most common cancer among newly diagnosed cancers worldwide and ranks second in cancer-related deaths among women [1]. Although several anticancer drugs have been introduced, breast cancer remains one of the leading causes of cancer-related deaths [1].

Platinum-based anticancer drugs (PBDs) are powerful broad-spectrum antitumor treatments effective against many solid tumors, including breast cancer. Cisplatin [cis-diammine-(dichloro)platinum(II)] is a first-generation platinum-based complex that was granted the US Food and Drug Administration (FDA) approval in the late ’70s. To date, hundreds of PBDs have been synthesized and entered clinical trials to enhance the anticancer activities and minimize toxic effects (including nephrotoxicity, ematogenesis, and resistance) compared to cisplatin. From these developed PBDs, only carboplatin and oxaliplatin have been approved by FDA for cancer treatment [2,3,4,5].

Oxaliplatin (1*R*,2*R*)-cyclohexane-1,2-diamine;oxalate;platinum(2+), a third-generation platinum derivative, has replaced cisplatin for the treatment of relapsed non-Hodgkin’s lymphoma, refractory ovarian cancer, non-small cell lung carcinoma, and metastatic breast cancer, as a more potent and safer drug [2,6]. Oxaliplatin forms intrastrand and interstrand DNA-platinum adducts, which interfere with gene transcription and/or cause G2/M stage arrest by segregating transcription factors [7,8,9]. Nevertheless, oxaliplatin has several shortcomings, such as systemic adverse reactions (caused by its reactivity toward thiol groups in plasma proteins), including peripheral distal neurotoxicity and acute dysesthesias [7,8,9]. Additionally, several clinical trials reported low efficiency of oxaliplatin monotherapy in treating some resistant tumors [2,6].

Hence, extensive efforts have been exerted to overcome the shortcomings of PBDs, including oxaliplatin. In this regard, PBDs have been reformulated into various delivery platforms to improve their selective uptake into target tumor cells while simultaneously minimizing off-target adverse effects [7]. Many delivery vehicles were employed, including liposomes, solid lipid nanoparticles, polymeric nanoparticles, and supramolecular host molecules [10,11,12,13,14,15,16]. The enhanced permeability and retention (EPR) effect, which results from leaky vasculature and poor lymphatic drainage within the tumor tissues, can effectively retain nanoparticles into tumor tissue [17].

Chitosan (CS), a semisynthetic polyaminosaccharide derived from *N*-deacetylated chitin, is biodegradable, biocompatible with exceptional physicochemical and mechanical properties [18,19,20]. CS NPs exhibit antioxidant, anticancer, and antibacterial activities [21]. Several studies have reported using CS as a carrier for different natural and synthetic chemotherapeutics [20]. Polyethylene glycol (PEG) modifying CS NPs is a promising nanoplatform for effective chemotherapeutics delivery [22]. This is attributed to the ability of the hydrophilic synthetic polymer to reduce the recognition of the NPs by the reticuloendothelial system (RES) thus prolonging their circulation time. Moreover, coating of CS NPs with PEG leads to their selective accumulation in the cancer site through enhanced permeability and retention (EPR) effect [22]. Previous studies reported the possible application of ascorbic acid (AA; vitamin C) in the cancer therapy [23]. It has been shown to promote apoptosis in human breast cancer cell lines without substantially affecting normal cells. Furthermore, AA is a powerful antioxidant that protects cellular components against the harmful effects of free radicals [24]. Currently, the use of AA clinically has been related to embracing new therapeutic approaches in which AA acts as a chemosensitizer for potential repurposed drugs. In this regard, pharmacological levels of AA were reported to be capable of sensitizing cancer cells to chemotherapeutic agents, augmenting their anticancer effects [25]. This synergism between AA and chemotherapeutics has been reported previously in many cancers including breast cancer [26,27]. Therefore, the possible augmenting effect of AA on OX when both compounds are encapsulated in CS NPs was investigated in this study.

In the present work, the fabrication of non-PEGylated and PEGylated chitosan nanoparticles (CS) loaded with ascorbic acid (AA), oxaliplatin (OX), or a combination of AA and OX was carried out using the ionotropic gelation method. The prepared nanoparticles were physically characterized in terms of average hydrodynamic diameters, zeta potential, polydispersity index (PDI), entrapment efficiency %, and morphology (utilizing transmission electron microscope). Furthermore, Fourier transform infrared spectroscopy (FTIR) was employed to study the chemical structural features of the nanoparticles, and thermal gravimetric analysis (TGA) was used to determine the organic contents. Additionally, the release profiles of AA, OX, and AA-OX from the non-PEGylated and PEGylated Cs NPs were evaluated at two pH values of 7.4 and 5.5. The prepared nanoparticles’ cytotoxicity was assessed against breast adenocarcinoma (MCF-7) cells using MTT assay and compared to free AA and OX. Finally, flow cytometry was carried out to analyze the mode of cell death in MCF-7 cells upon treatment with the prepared nanoparticles compared to the unloaded drugs.

## 2. Materials and Methods

### 2.1. Materials

Oxaliplatin was obtained from BLD Pharmatech Co., Limited, Cincinnati, OH, USA, l-ascorbic acid (MWt = 176.12 g/mol) and PEG 400 were purchased from Sigma-Aldrich, St. Louis, MO, USA. Low molecular weight chitosan was purchased from Biosynth, Carbosynth, Berkshire, UK. TPP (sodium tripolyphosphate) was purchased from Advent, Navi Mumbai, India. Dulbecco’s modified Eagle’s medium (DMEM) with 4.5 g/L glucose, 0.05% Trypsin and Phosphate-Buffered Saline (PBS, pH 7.4), DMEM without l-Glutamine or phenol red and Trypan Blue with 0.85% NaCl were purchased from Lonza Bioscience (Walkersville, MD, USA). Dimethyl sulfoxide (DMSO) was obtained from Serva (Heidelberg, Germany). Annexin V-FITC/PI Apoptosis Detection Kit was purchased from Elabscience (Wuhan, China). Ethanol and 3-(4,5-dimethylthiazol-2-yl)-2,5-diphenyltetrazolium bromide (MTT) were obtained from ThermoFisher Scientific (Waltham, MA, USA). Fetal Bovine Serum (FBS) was obtained from Gibco (Waltham, MA, USA). MCF-7 cell lines (Cat No. HTB-22) was purchased from ATCC (Manassas, VA, USA).

### 2.2. Methods

#### 2.2.1. Preparation of the NPs

Drug-loaded chitosan NPs were prepared using the ionotropic gelation method, as previously reported, with some modifications [28,29]. Briefly, 1 mg/mL CS aqueous solution containing 2% (*v*/*v*) glacial acetic acid was prepared, and the pH was adjusted to 4 using 10 M NaOH solution and filtered with syringe filters of 0.45 µm pores sized filters. Then, the solution was left on a magnetic stirrer for 24 h. Afterward, 200 μL of the crosslinker sodium tripolyphosphate (1 mg/mL, TPP) was mixed with AA, OX, or AA-OX and then added dropwise to 5 mL of CS solution under magnetic stirring for 30 min. This generated AA/CS NPs, OX/CS NPs, and AA-OX/CS NPs, respectively.

PEGylated CS NPs (PEG-CS NPs) were prepared by adding 250 µL of PEG 400 dropwise to 5 mL of chitosan solution under magnetic stirring for 30 min forming a homogeneous mix of chitosan and PEG. Then 200 µL of sodium tripolyphosphate (1 mg/mL, TPP) crosslinker was mixed with AA, OX, or AA-Ox and then added dropwise to CS/PEG mixture solutions under magnetic stirring for 30 min. This produced AA/PEG-CS NPs, OX/PEG-CS NPs, and AA-OX/PEG-CS NPs.

#### 2.2.2. Characterization of the Prepared NP

The hydrodynamic diameter (HD) and polydispersity index (PDI) of non-PEGylated and PEGylated CS NPs were determined using dynamic light scattering employing Zetasizer Nano ZS (Malvern Instruments, Herrenberg, Germany). All measurements took place at 25 °C, where water’s refractive index and viscosity were 1.33 and 0.887 mPa·s, respectively. The instrument was equipped with a 10 mW HeNe laser allowing for the measurements to be performed at a wavelength of 633 nm and a detection angle of 173° backscatter. **ζ**-potential was measured utilizing laser Doppler velocimetry in a clear disposable folded capillary cell (DTS1070, Malvern Instruments). All measurements took place in triplicates, and standard deviation (SD) of three independent measurements was calculated [30].

The NPs’ morphology was investigated employing transmission electron microscopy (TEM) using a JEOL-JEM 2100 electron microscope (Musashino, Akishima, Tokyo, Japan) operating at 160 kV. NPs (a 50 µL aliquot of each sample were diluted with deionized water in the ratio of 2:1 (*v*/*v*). Afterward, 2% aqueous phosphotungstic acid was employed to stain the diluted NPs. This mixture was added dropwise and dried over a carbon-coated copper 200 mesh grid, imaged, and photographed [31].

The FTIR spectra of AA, OX, CS NPs, PEGylated CS NPs, AA/CS NPs, OX/CS NPs, AA-OX/CS NPs, AA/PEG-CS NPs, OX/PEG-CS NPs, and AA-OX/PEG-CS NPs were acquired using FTIR spectroscopy (FTIR-8400s, Shimadzu, Kyoto, Japan). Pellets were produced by compressing the samples with KBr under hydraulic pressure, scanned, and spectra were obtained in the range of 4000−500 cm^−1^ [31].

#### 2.2.3. Quantification Method

An ultra-performance liquid chromatography system (UPLC Agilent ultra-performance 1290 infinity, Agilent Technologies, Santa Clara, CA, equipped with 1290 DAD, gradient quaternary pump VL, auto-sampler ALS, column oven TCC and 1290 Thermostat, has been used to quantify AA, OX, and AA/OX in the non-PEGylated and PEGylated CS NPs. A C18 column (InfinityLab Poroshell 120; 3.0 × 150 mm, 1.9 µm, was used, and the injection volume was 1 µL. The mobile phase consisted of 0.1% Triethylamine in water, and the pH was adjusted to 4.0 using acetic acid and acetonitrile (95:5, *v*/*v*). Analysis was performed at a column temperature of 20 °C, and detection was carried out using a diode array detector (DAD) 280 nm. Data acquisition was performed on Agilent Chemstation software (B.04.03) and data processing was subsequently performed using Agilent LabAdvisor Quantitative analysis software (B.02.04). Separation was completed in less than 7 min with retention times of 3.3 and 6.1 min for AA and OX, respectively. The method was validated by carrying out linearity, specificity, accuracy, and precision studies (see Supplementary Materials).

#### 2.2.4. Determination of Entrapment Efficiency % (EE) of Ascorbic Acid and Oxaliplatin in Chitosan Nanoparticles

The EE % of AA/CS NPs, OX/CS NPs, AA-OX/CS NPs, AA/PEG-CS NPs, OX/PEG-CS NPs, and AA-OX/PEG-CS NPs was carried out as described elsewhere, with some modifications [12,29]. Briefly, 2 mL of each sample was centrifuged (15,000 rpm, 2 h, 4 °C); (Hermle Z 326 K, Labortechnik GmbH, Wehingen, Germany). Then, the supernatant of the formulation was ultrafiltrated, and the unencapsulated AA, OX, and AA-OX were quantified using HPLC, as described in Section 2.2.3. The EE (%) of the AA/CS NPs, OX/CS NPs, AA-OX/CS NPs, AA/PEG-CS NPs, OX/PEG-CS NPs, and AA-OX/PEG-CS NPs was determined using Equation (1) [12,32,33].

(1) $$\mathrm{EE}\;\%=\frac{\mathrm{Total}\;\mathrm{amount}\;\mathrm{of}\;\mathrm{drug}-\mathrm{the}\;\mathrm{amount}\;\mathrm{of}\;\mathrm{drug}\;\mathrm{unencapsulated}}{\mathrm{Total}\;\mathrm{amount}\;\mathrm{of}\;\mathrm{drug}}\times 100$$

#### 2.2.5. Release Study

AA and OX release rates from AA/CS NPs, OX/CS NPs, AA-OX/CS NPs, AA/PEG-CS NPs, OX/PEG-CS NPs, and AA-OX/PEG-CS NPs were investigated utilizing the dialysis membrane technique at pH 5.5. Briefly, 0.5 mL of the sample was loaded in a dialysis bag (12–14 KD cut off) placed in 25 mL PBS at pH values of 7.4 and 5.5. The container was placed in a shaking incubator (Jeio tech SI-300, Seoul, Korea), rotating at 100 rpm and 37 ± 0.5 °C. At specific time intervals, 1 mL aliquots of the sample were collected for quantification by HPLC (described in Section 2.2.3) and immediately replaced with another 1 mL of warmed buffer. All experiments were performed in triplicates, and standard deviation (SD) was calculated.

#### 2.2.6. Cell Viability Assay

##### Cell Culture

MCF-7 cells (HTB-22; ATCC, Manassas, VA, USA) were maintained in DMEM supplemented with 5% penicillin-streptomycin, and 10% FBS and incubated at 37 °C and 5% CO_2_. Cells were stained with Trypan blue and viable cell count determined using a hemocytometer. For the MTT assay, MCF-7 cells were seeded in 96-well plates at a seeding density of 10,000 cells/well.

##### MTT Assay

MCF-7 cells were seeded in 96-well plates for 24 h before treatment with increasing concentrations of different compounds or vehicles for an additional 48 h. At the end of the treatment, the supernatant was discarded, and 20 μL of (5 mg/mL) MTT solution was added in addition to 80 μL complete media (total of 100 μL) for 3 h. The supernatant was discarded and replaced with 100 μL 100% DMSO to dissolve formazan crystals, followed by measurement of absorbance at 570 nm using a microtiter plate reader. MCF-7 cell viability was calculated = A (sample)/A (control) × 100. For all experiments, control groups were MCF-7 cells cultured in serum-free media treated with vehicle. The absorbance data at 570 nm was plotted against different concentrations of each compound to calculate the viability inhibition concentration at 50% (IC_50_) values using GraphPad Prism 9.0 (San Diego, CA, USA). The experiment was repeated in quadruplicates.

#### 2.2.7. Flow Cytometry and Cell Apoptosis Assay

MCF-7 cells were seeded at a density of 500,000 cells per flask in T-25 flasks and incubated for 24 h at 37 °C with 5% CO_2_. MCF-7 cells were exposed to different treatments or vehicles separately in T-25 flasks. GraphPad Prism was used to compute IC_50_ values for the various drugs based on the findings of the cell viability assay. As a result, the IC_50_ values were employed to treat MCF-7 cells. After 48 h of treatment, cells were prepped and sampled in preparation for flow cytometry measurements. Initially, MCF-7 cells were washed in PBS before being trypsinized and incubated for 5 min. Complete DMEM medium was then added, and the cell suspension was transferred to 15 mL tubes. The treated MCF-7 cells were then pelleted by centrifugation at 125× *g* for 7 min. The supernatant was discarded, the pelleted MCF-7 cells were washed again with PBS, and the supernatant was removed after centrifugation at 300× *g* for 5 min. Subsequently, 1 mL PBS was added to the control group’s falcon tubes alone to re-suspend the pellet before centrifugation. To resuspend the pellet, 500 μL of annexin V binding buffer was added to all groups. For the preparation of 1X annexin V binding buffer, 900 μL of 9X annexin V binding buffer were added to 8100 μL of deionized water. The PI dye was then made by mixing 5 μL of PI with 45 μL of annexin V binding buffer. After that, the cells were stained with 1 μL of PI dye and 5 μL of annexin V dye. Before the measurements, the samples were incubated in the dark for 15 min and 400 μL of 1X annexin V binding buffer was added to each sample. MCF-7 cell apoptosis was assessed using a flow cytometer (Attune™ NxT, ThermoFisher Scientific, Waltham, MA, USA). The experiment was repeated twice. The obtained data were analyzed using FlowJo software Version 10.6.2 (Ashland, OR, USA).

## 3. Results and Discussion

### 3.1. Hydrodynamic Diameter, Polydispersity Index (PDI), ζ-Potential, Entrapment Efficiency (EE%) and Morphology

The hydrodynamic diameter and PDI of the non-PEGylated and PEGylated CS NPs were studied utilizing dynamic light scattering, and the results are summarized in Table 1 and Table 2. The diameters of all prepared nanoparticles were found to lie in the range between 150−300 nm previously reported for different nanosystems encapsulating anticancer drugs. This size range facilitates the passive accumulation of the drugs into tumor tissues with leaky vasculature and poor lymphatic drainage [34,35]. As presented in Table 1, the ζ-potential of the fabricated nanoparticles demonstrated high positive surface charges (due to cationic CS) of +27.60 ± 1.48, +22.02 ± 1.50, +22.58 ± 1.85 and +40.40 ± 2.71 mV for CS NPs, AA/CS NPs, OX/CS NPs, and AA-OX/CS NPs, respectively. These high positive surfaces prevent the aggregation of the NPs and improve their stability. Entrapment efficiencies of the three prepared nanoparticles are presented in Table 1, and show the potential of CS NPs to entrap high concentrations of either AA, OX, or AA-OX. Dual drug-loaded CS NPs with AA and OX had a noticeable effect on the particle size, ζ-potential, and EE% of either AA or OX. A remarkable increase in the particle size, ζ-potential, and EE% was observed upon the co-loading of AA and OX.

Previous studies reported the improvement in the physicochemical and biological properties of CS NPs upon coating their surface with PEG [28,36].

As presented in Table 2, coating of CS NPs with PEG has reduced the diameters and PDI. Likewise, the ζ-potential of the PEGylated CS NPs has been reduced owing to the shielding of the positive charges by the PEG chains. During the crosslinking of chitosan, PEG builds an interpenetrating structural network with chitosan, increasing the surface compactness of CS NPs, leading to a reduction in diameter and surface charges after PEGylation [28].

On the other hand, the EE% of either AA or OX had been improved in the case of AA/PEG-CS NPs, OX/PEG-CS NPs, and AA-OX/PEG-CS NPs. This is attributed to the colloidal stabilization effect caused by PEG chains on the surfaces of the CS NPs [28].

**Figure 1:**
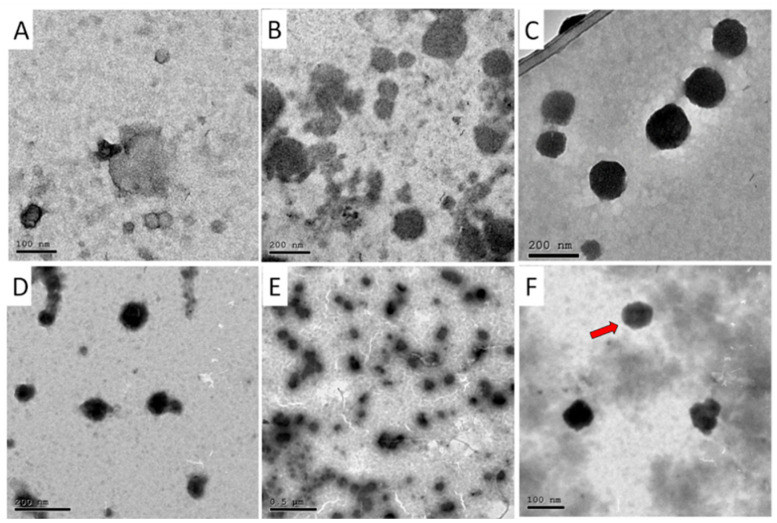
TEM images for (**A**) AA/CS NPs, (**B**) OX/CS NPs, (**C**) AA-OX/CS NPs, (**D**) AA/PEG-CS NPs, (**E**) OX/PEG-CS NPs, and (**F**) AA-OX/PEG-CS NPs. The arrow shows the PEG layer coating the CS NPs.

The TEM images of the non-PEGylated and PEGylated CS NPs are demonstrated in Figure 1. TEM analysis revealed the successful formation of spherical NPs with smooth surfaces for AA/CS NPs, OX/CS NPs, and AA-OX/CS NPs. Furthermore, TEM analysis showed that coating the CS NPs with PEG has not affected their spherical morphologies.

### 3.2. Fourier-Transform Infrared Spectroscopy (FTIR)

**Figure 2:**
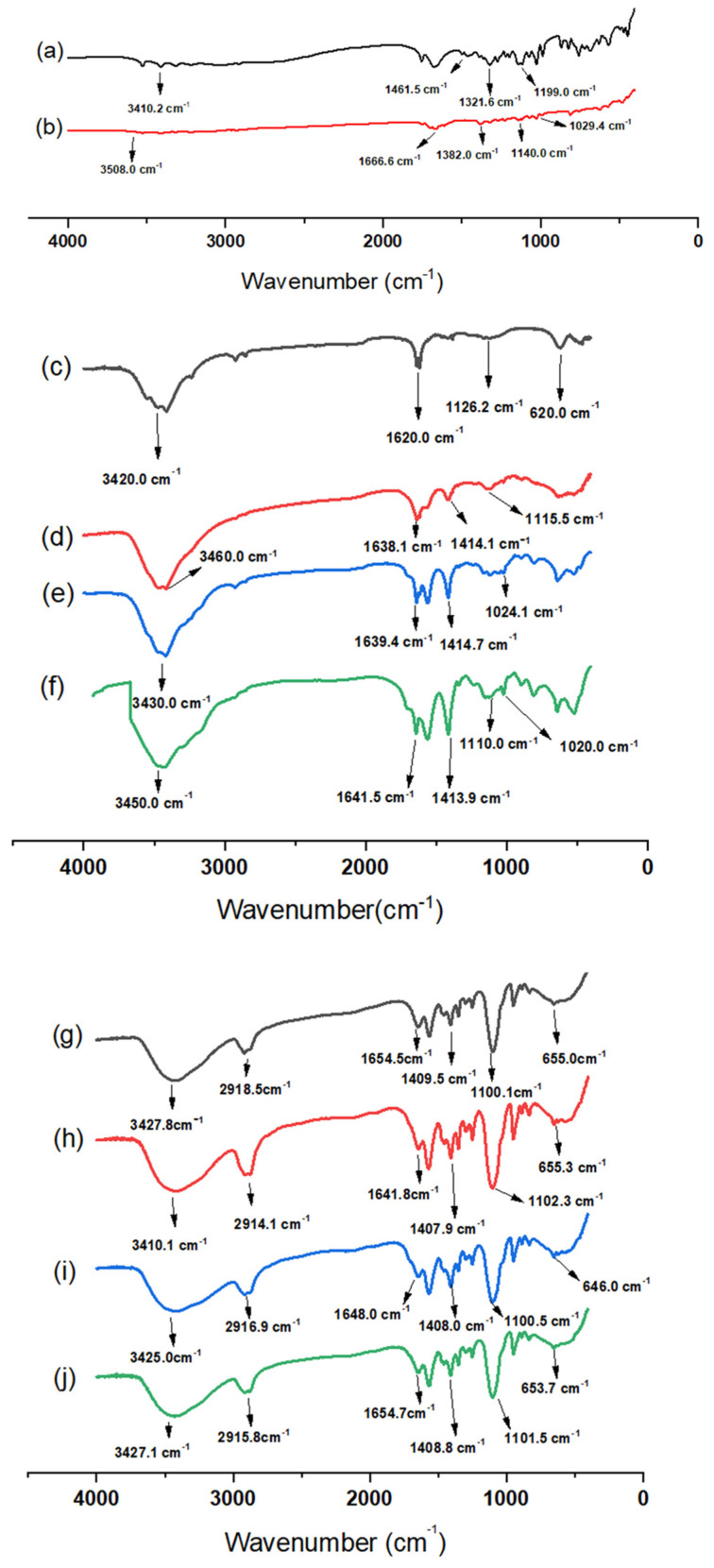
FTIR spectra of (**a**) pure ascorbic acid, (**b**) pure oxaliplatin, (**c**) unmodified CS NPs, (**d**) AA/CS NPs, (**e**) OX/CS NPs, (**f**) AA-OX/CS NPs, (**g**) PEG-CS NPs, (**h**) AA/PEG-CS NPs, (**i**) OX/PEG-CS NPs, and (**j**) AA-OX/PEG-CS NPs.

FTIR analysis data revealed various characteristic peaks (Figure 2)**.** The FTIR spectrum of AA showed four major peaks at 3410 cm^−1^ (–OH stretching), 1416 cm^−1^, 1321cm^−1^ (–CH bending), and 1199 cm^−1^ (C–O–C stretching) [37,38]. Also, the FTIR spectrum of OX showed four characteristic peaks at 3508 cm^−1^ (–NH), 1666 cm^−1^ (C=O stretching), 1382 cm^−1^ (–CH bending) and 1140 cm^−1^ (C–O–C) [39]. The FTIR spectrum of CS NPs showed distinct peaks at 3420 and 1620 cm^−1^, which may correspond to stretching vibrations of amine (–NH_2_) and/or hydroxyl (–OH) and (C=C bond) and carboxylic (C=O bond) groups, respectively. Additionally, one peak was observed at 1126 cm^−1,^ corresponding to an alcoholic (C–O) stretching vibration [40]. On the other hand, the FTIR spectra of AA/CS NPs, OX/CS NPs, and AA-OX/CS NPs showed all major peaks of AA, OX, and CS NPs with no remarkable shifts, suggesting the physical entrapment of the drug/s within the CS matrix [41]. The FTIR spectra of PEGylated CS NPs (Figure 2g) revealed two characteristic peaks at 1409 cm^−1^ (–CH bending) and 1100 cm^−1^ (C–O–C stretching) [42]. Additionally, one peak was detected at 2918 cm^−1,^ which may be attributed to –CH_2_ stretching vibration. This indicates the successful coating of CS NPs with PEG [43]. The FTIR spectra of AA/PEG-CS NPs, OX/PEG-CS NPs, and AA-OX/PEG-CS NPs (Figure 2h–j) revealed the presence of all main AA, OX, and PEGylated CS NPs peaks implying physical entrapment of the drug/s inside the CS matrix. These findings are in the same line with previous studies that reported the fabrication of nanocarriers coated with PEG [42,43].

### 3.3. Release Study

**Figure 3:**
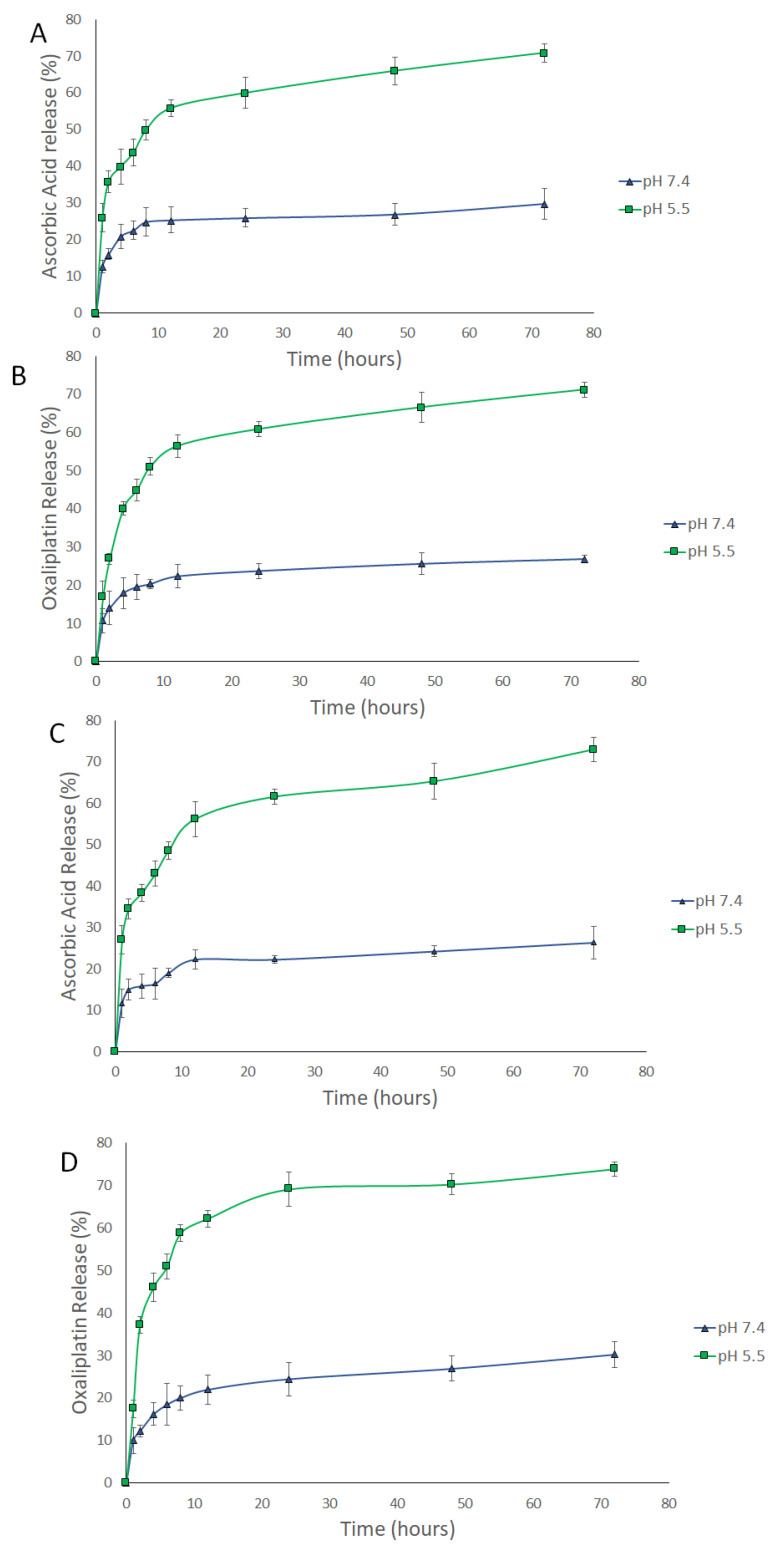
Time-dependent release profiles of (**A**) AA from AA/CS NPs, (**B**) OX from OX/CS NPs, (**C**) AA from AA-OX/CS NPs and (**D**) OX from AA-OX/CS NPs at 37 °C, at pH 7.4 and 5.5.

**Figure 4:**
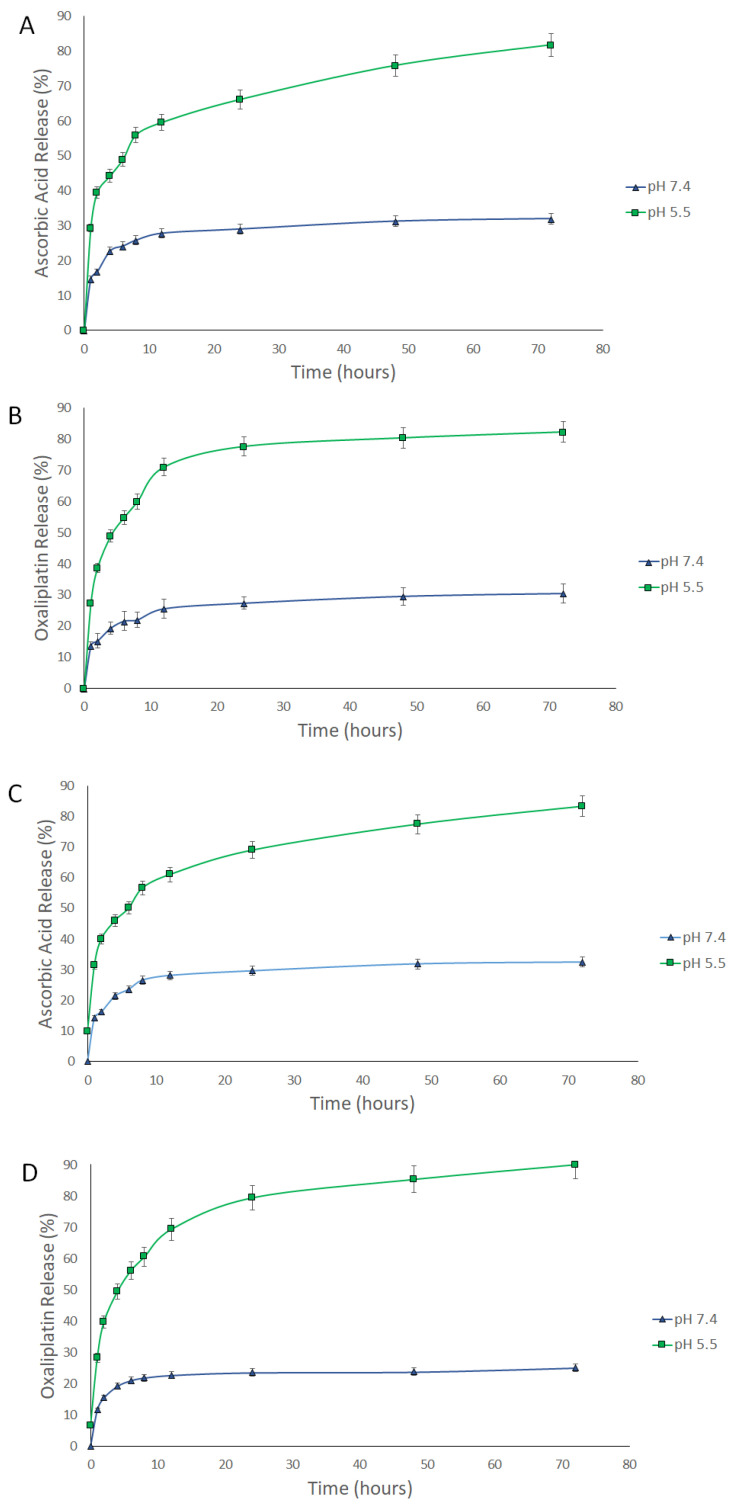
Time-dependent release profiles of (**A**) AA from AA/PEG-CS NPs, (**B**) OX from OX/PEG-CS NPs, (**C**) AA from AA-OX/PEG-CS NPs, and (**D**) OX from AA-OX/PEG-CS NPs at 37 °C, at pH 7.4 and 5.5.

The release of AA, OX, and AA-OX from the chitosan NPs was studied at pH 7.4 (healthy cell microenvironment), and pH 5.5 (cancer cells’ microenvironment) (Figure 3 and Figure 4). The released drugs were determined utilizing the UHPLC method described in Section 2.2.3. The non-PEGylated and PEGylated CS NPs exhibited outstanding stability at pH 7.4. In the case of non-PEGylated CS NPs (Figure 3), about 29.8% and 26.8% of the loaded AA and OX were released after 72 h from AA/CS NPs and OX/CS NPs, respectively. Also, 26.38% and 30.21% of the loaded AA and OX were released after 72 h from AA-OX/CS NPs after 48 h at 37 °C. In the case of PEGylated CS NPs (Figure 4), about 31.96% and 30.48% of the loaded AA and OX were released after 72 h from AA/PEG-CS NPs and OX/PEG-CS NPs, respectively. While about 32.43% and 25.07% of the loaded AA and OX were released from AA-OX/PEG-CS NPs after 72 h at 37 °C.

Both non-PEGylated and PEGylated CS NPs showed much faster release rates in the acidic medium than pH 7.4. In the case of non-PEGylated CS NPs, 70% and 71% of the loaded AA and OX were released after 72 h (at pH 5.5 and 37 °C) from AA/CS NPs and OX/CS NPs, respectively. On the other hand, 73% and 74% of the loaded AA and OX were released from AA-OX/CS NPs after 72 h. In the case of PEGylated CS NPs, 81.8% and 82.2% of the incorporated AA and OX were released after 72 h from AA/PEG-CS NPs and OX/PEG-CS NPs, respectively. On the other hand, about 83.2% and 90% of the loaded AA and OX were released after 72 h from AA-OX/PEG-CS NPs. These results showed that coating CS NPs with PEG did not hinder the release of the drugs from CS NPs in the acidic environment Protonation of CS’s amino groups occurs in the acidic medium thus increasing the repulsive electric forces between the crosslinked CS chains. This leads to the swelling of the CS NPs by permitting the medium to penetrate the CS NPs leading to the faster diffusion of the drugs from CS NPs [44,45,46]. The enhanced release of AA and OX at the pH of 5.5 would eventually improve the anticancer potency of the designed nanocarriers against breast cancer cells.

### 3.4. Cell Viability Assay

**Figure 5:**
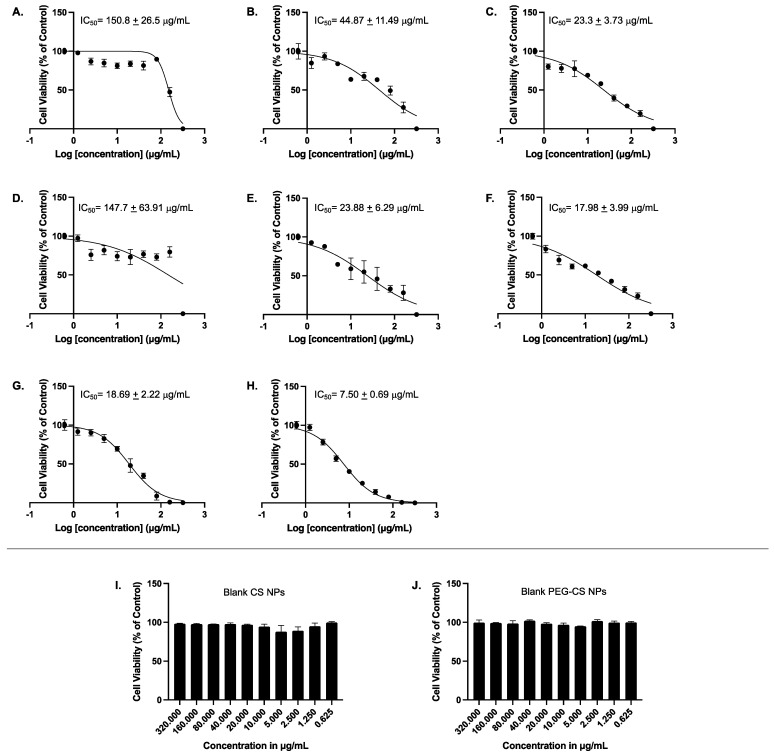
Concentration-dependent effects of the tested compounds on cellular viability of MCF-7 cells. MCF-7 cells were treated with 10 increasing concentrations (0.625, 1.25, 2.5, 5, 10, 20, 40, 80, 160, and 320 μg/mL) of AA (**A**), AA/CS NPs (**B**), AA/PEG-CS NPs (**C**), OX (**D**), OX/CS NPs (**E**), OX/PEG-CS NPs (**F**), AA-OX/CS NPs (**G**), AA-OX/PEG-CS NPs (**H**), blank CS NPs (**I**), or blank PEG-CS NPs (**J**) for 48 h. Thereafter cellular viability was measured using MTT assay as described in Section 2.2.6. The experiment has been conducted in triplicates. It should be noted that IC50 value in 5D (147.7 µg/mL) which apparently does not fall in the range of 160 to 320 µg/mL is a visual error which can be attributed to the differences between relative and absolute IC50 calculations. That is the IC50 value reported in the current figure is the relative IC50 value and not the absolute one. Pharmacologically, the relative IC50 calculation method is the common one used to report IC50 values. In addition, the curve fitting approach takes into consideration the maximal and minimal responses and not simply the values between two adjacent concentrations.

MCF-7 cells were treated for 48 h with 10 increasing concentrations (0.625, 1.25, 2.5, 5, 10, 20, 40, 80, 160, and 320 μg/mL) of AA, AA/CS NPs, AA/PEG-CS NPs, OX, OX/CS NPs, OX/PEG-CS NPs, AA-OX/CS NPs, AA-OX/PEG-CS, in addition to blank non-PEGylated and blank PEGylated NPs; thereafter cellular viability was assessed using MTT assay. Figure 5A shows that AA alone had an IC_50_ value of 150.8 ± 26.5 µg/mL. Interestingly, when AA was encapsulated into CS NPs, the formulation had a lower IC_50_ value of 44.87 ± 11.49 µg/mL (Figure 5B). OX alone had an IC_50_ value of 147.7 ± 63.91 µg/mL (Figure 5D). Importantly, when OX was encapsulated into CS NPs, it was more potent in reducing cellular viability, with its IC_50_ value being 23.88 ± 6.29 µg/mL (Figure 5E). When both AA and OX were encapsulated separately into PEG-CS NPs, there was further potentiation to their abilities to reduce cellular viability with an IC_50_ value of 23.3 ± 3.73 and 17.98 ± 3.99 µg/mL, respectively (Figure 5C,F). It was also of importance to test the combination of AA and OX in CS NPs in addition to CS-PEG NPs on MCF-7 cells. AA-OX/CS NPs exhibited an IC_50_ value of 18.69 ± 2.22 µg/mL while AA-OX/CS-PEG NPs had the lowest IC_50_ value reaching 7.50 ± 0.69 µg/mL. Lastly, and to ensure that the effect of the NPs encapsulated AA and OX is not attributed to the polymers alone, we sought to determine the effect of the blank CS NPs and blank PEG-CS NPs on MCF-7 cellular viability. Our results demonstrated that none of the tested concentrations of the blank NPs had any significant effect on MCF-7 cellular viability, eliminating the possibility that the effect of encapsulated AA or OX is due to the cytotoxic effects of the blank NPs alone on MCF-7 cells (Figure 5I,J). Therefore, the cytotoxicity of AA and OX encapsulated in CS-NPs and PEG-CS NPs is not due to any cytotoxic effects of the blank NPs. Our results are in agreement with previously published studies showing that blank chitosan NPs had little to no effect on the cellular viability of different cell lines [47,48].

The high positive charge on the surface of NPs enhances their binding to the negatively charged cancer cell membranes [49]. The combination of AA and OX yielded an enhanced anticancer activity that might be attributed to either synergistic or additive effects [50]. Furthermore, CS NPs and CS-PEG NPs encapsulating AA and OX seem to would have facilitated the EPR effect leading to the passive targeting of loaded drugs into tumor tissues. The designed nanoparticles also protect their cargos from systemic undesired side reactions and hence, further improve the accumulation of AA and OX inside cancer cells. In a clinical setting, AA serves as a chemosensitizer. While some reports suggest it may protect cancer cells from chemotherapy, others claim that pharmaceutical doses of AA might make cancer cells more sensitive to chemotherapy, improving its anticancer effect. In this regard, several studies have reported the synergistic effect of AA with conventional chemotherapeutic drugs in various types of cancer, including pancreatic [51], prostate [52], lung [53], breast [26,27], and ovarian [54]. In agreement with these studies, our results have confirmed a chemosensitizing effect of AA to OX treatment in the MCF-7 breast cancer cells. The presence of AA together with OX in either unmodified CS or CS-PEG NPs formulations resulted in lower IC_50_ values compared to each of them separately. Encapsulation of OX in pegylated multi-walled carbon nanotubes significantly decreased cellular viability as compared to that of non-PEGylated nanotubes encapsulating OX [55]. Our results demonstrate that PEGylation does indeed lower the IC_50_ values of both AA and OX upon encapsulation in CS-PEG NPs either individually or combined, which could in part be attributed to the higher encapsulation efficiency of the drugs into the PEGylated formulations (Table 1 and Table 2). 

### 3.5. Flow Cytometry and Cell Apoptosis Assay 

**Figure 6:**
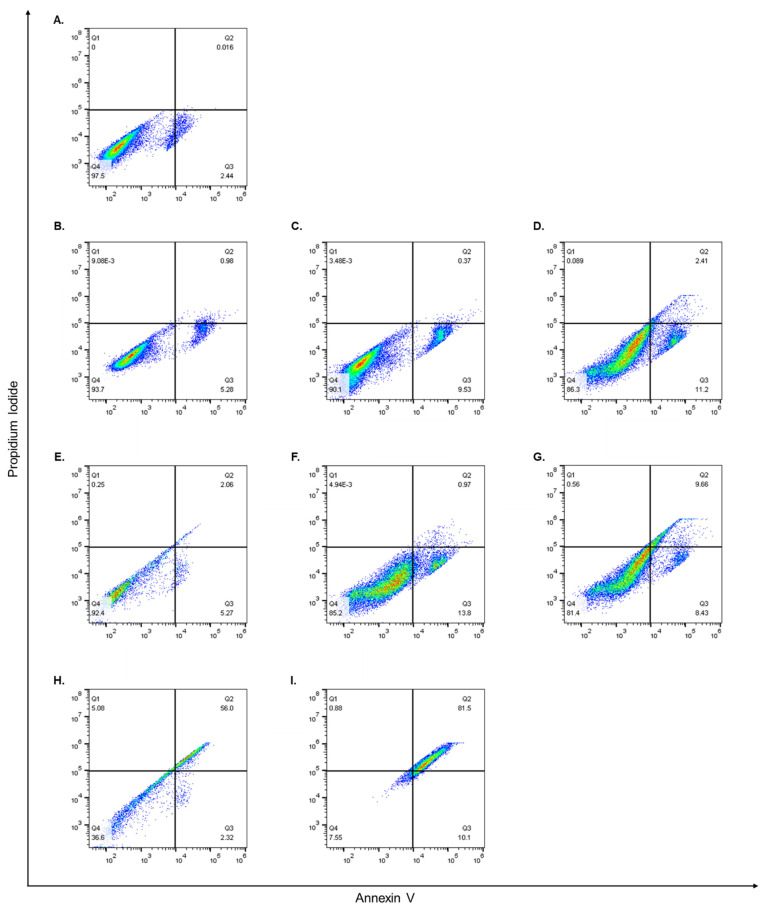
Apoptotic effects of free and encapsulated AA and OX. MCF-7 cells were treated for 48 h with CS NPs (**A**) or the IC_50_ concentrations of AA (**B**), AA/CS NPs (**C**), AA/PEG-CS NPs (**D**), OX (**E**), OX/CS NPs (**F**), OX/PEG-CS NPs (**G**), AA-OX/CS NPs (**H**) or AA-OX/PEG-CS NPs (**I**). Thereafter, the percentages of viable, early, and late apoptotic cells were detected via Annexin/PI staining that was measured by the flow cytometry analysis, as described in Section 2.2.7. The experiment has been conducted in duplicates.

While it was evident that encapsulating both AA and OX alone or together in CS or PEG-CS NPs improved their cytotoxic effects on MCF-7 cells, it was important to examine their apoptotic effects. Therefore, MCF-7 cells were treated for 48 h with the IC_50_ concentrations determined by the MTT assay, and thereafter, the percentage of apoptotic cells was detected using Annexin/PI staining that was measured by flow cytometry analysis, as previously described [56]. Our results showed that cells treated with the different compounds had differential effects on early and late apoptosis in MCF-7 cells. AA alone induced apoptosis, and the percentages of viable cells, early apoptotic, and late apoptotic cells were 93.7%, 5.28%, and 0.98% (Figure 6B) compared to 97.5%, 2.44%, and 0.01%, respectively, in control cells (Figure 6A). AA/CS NPs showed an enhanced early apoptotic effect compared to AA alone as the percentage of viable cells, early apoptotic, and late apoptotic cells were 90.1%, 9.53%, and 0.37%, respectively (Figure 6C). Intriguingly, when cells were treated with AA/CS-PEG NPs, there was a further increase in apoptotic cells compared to AA/CS NPs, and the percentages of viable cells, early apoptotic, and late apoptotic cells were 86.3%, 11.2%, and 2.41%, respectively (Figure 6D). OX alone also induced apoptosis, and the percentages of viable cells, early apoptotic, and late apoptotic cells were 92.4%, 5.27%, and 2.06%, respectively (Figure 6E). OX/CS NPs showed an enhanced early apoptotic effect compared to OX alone as the percentage of viable cells, early apoptotic, and late apoptotic cells were 85.2%, 13.8%, and 0.97%, respectively (Figure 6F). Similar to AA, when cells were treated with OX/CS-PEG NPs there was a further increase in apoptotic cells compared to OX/CS NPs, and the percentages of viable cells, early apoptotic, and late apoptotic cells were 81.4%, 8.43%, and 9.66%, respectively (Figure 6G). Moreover, treating MCF-7 cells with AA-OX/CS NPs depicted a fall in the percentage of viable cells reaching 36.6%, accompanied by a rise in the percentages of early and late apoptotic cells reaching 2.32% and 56%, respectively (Figure 6H). Additionally, when MCF-7 cells were treated with AA-OX/CS-PEG NPs, the percentage of viable cells went down to 7.55%, with an increase in early and late apoptotic cells reaching 10.1% and 81.5%, respectively (Figure 6I). Annexin V binds to phosphatidylserine on the cell membrane’s extracellular surface. PI on the other hand, attaches to DNA, however, it can only get into cells with a damaged membrane. In addition, PI will not stain live cells with an unbroken cell membrane. In toto, cells that are positive for both Annexin V and PI are regarded as late apoptotic/dead, while those that are just positive for Annexin V are thought to be early apoptotic/dying [57,58].

Apoptosis has multiple complex and sophisticated mechanisms involving an energy-dependent cascade of molecular reactions. There are two main apoptotic mechanisms: the extrinsic or death receptor pathway and the intrinsic or mitochondrial pathway. In previous studies conducted on the LS1034 colorectal carcinoma cell line, AA has been shown to stimulate the intrinsic mechanism of apoptosis, whereas OX can activate the extrinsic pathway [59,60]. However, there is now evidence that the two routes are intertwined and that molecules in one can influence molecules in the other [61]. Our results demonstrated that MCF-7 cells treated with AA/CS NPs alone had more early apoptotic cells than those of control cells and that encapsulating AA in CS NPs enhanced the percentage of early apoptotic cells, and PEGylation caused further enhancement to the early apoptotic cell percentages. OX alone had a higher overall percentage of apoptotic cells (early and late), compared to AA alone. Additionally, when OX was encapsulated in CS NPs there was an increase in the percentage of apoptotic cells that were further enhanced by PEGylation of the CS NPs. Importantly, when cells were treated with AA-OX/CS NPs there was a sharp increase in the number of apoptotic cells compared to any of the previous treatments and this was further potentiated upon the PEGylation of the CS NPs. These results strongly support the fact that encapsulating AA and OX into CS NPs enhances their apoptotic effect and that PEGylation of these NPs potentiates their killing effect. Additionally, combining AA and OX in CS NPs or CS-PEG NPs exacerbates their apoptotic effects. In case of CS-PEG NPs, PEG enhances the entry of NPs through the cellular membrane and prevent its cargo from immature release before entering the cell. 

## 4. Conclusions 

Non-PEGylated and PEGylated CS NPs encapsulating AA and/or OX were prepared by the ionic-gelation method. The PEGylated CS NPs showed smaller average particle sizes, PDI, ζ-potentials, but more EE%. Release studies showed the capability of the prepared non-PEGylated and PEGylated chitosan NPs to release their cargo at the acidic environment of cancer tissue (pH 5.5) while maintaining stability at physiological pH of 7.4. The AA-OX/PEG-CS NPs exhibited the highest cytotoxic activities against breast adenocarcinoma (MCF-7) cells. Additionally, the AA-OX/PEG-CS NPs showed the highest percentages of early and late apoptotic MCF-7 cells. Taken together, these results demonstrate that PEGylated CS NPs encapsulating AA and OX individually or together could be a promising strategy for breast cancer treatment. Additionally, AA may have chemosensitized MCF-7 cells to OX treatment, which warrants further investigations to test the effects of combinations of other chemotherapeutics with AA.

## Figures and Tables

**Table 1:** Characterization of unmodified CS NPs, AA/CS NPs, OX/CS NPs and AA-OX/CS NPs. Data are means ± SD (*n* = 3).

Samples	Diameter (nm)	PDI	ζ-Potential (mV)	EE(%)	
				AA	OX
CS NPs	290.30 ± 7.45	0.41 ± 0.009	+27.60 ± 1.48	-	-
AA/CS NPs	157.20 ± 2.40	0.31 ± 0.04	+22.02 ± 1.50	75.49 ± 1.94	-
OX/CS NPs	188.10 ± 9.70	0.25 ± 0.06	+22.58 ± 1.85	-	78.73 ± 2.17
AA-OX/CS NPs	261.10 ± 9.19	0.43 ± 0.03	+40.40 ± 2.71	77.52 ± 2.35	79.25 ± 3.93

**Table 2:** Characterization of PEG-CS NPs, AA/PEG-CS NPs, OX/PEG-CS NPs, and AA-OX/PEG-CS NPs. Data represent means ± SD (*n* = 3).

Samples	Diameter (nm)	PDI	ζ-Potential (mV)	EE (%)	
				AA	OX
PEG-CS NPs	273.30 ± 2.69	0.30 ± 0.07	+15.73 ± 0.98	-	-
AA/PEG-CS NPS	152.20 ± 2.40	0.31 ± 0.04	+21.84 ± 1.54	90.92 ± 1.19	-
OX/PEG-CS NPs	156.60 ± 4.82	0.24 ± 0.03	+21.31 ± 1.78	-	94.11 ± 1.98
AA-OX/PEG CS NPs	176.00 ± 4.21	0.26 ± 0.04	+28.23 ± 0.93	91.84 ± 1.03	95.30 ± 1.49

## Data Availability

The data presented in this study are available upon request from the corresponding author.

## Supplementary Materials

The following supporting information can be downloaded at: https://www.mdpi.com/article/10.3390/pharmaceutics14020407/s1, Figure S1: Calibration curves of (a) AA, (b) OX over the concentration range of 5–100 µg/mL, Figure S2: UHPLC chromatogram of 1 µL injection of (a) AA (500 μg/mL), (b) OX (1000 μg/mL) and (c) Lab prepared mixture of AA and OX (100 μg/mL).
