# Correction: Fahmy et al. PEGylated Chitosan Nanoparticles Encapsulating Ascorbic Acid and Oxaliplatin Exhibit Dramatic Apoptotic Effects against Breast Cancer Cells. *Pharmaceutics* 2022, *14*, 407

**DOI:** 10.3390/pharmaceutics18040511

**Published:** 2026-04-21

**Authors:** Sherif Ashraf Fahmy, Asmaa Ramzy, Asmaa A. Mandour, Soad Nasr, Anwar Abdelnaser, Udo Bakowsky, Hassan Mohamed El-Said Azzazy

**Affiliations:** 1 Department of Chemistry, School of Sciences & Engineering, The American University in Cairo, AUC Avenue, P.O. Box 74, New Cairo 11835, Egypt; sheriffahmy@aucegypt.edu (S.A.F.); asmaaramzy95@aucegypt.edu (A.R.); 2 Pharmaceutical Chemistry Department, Faculty of Pharmacy, Future University in Egypt, Cairo 11835, Egypt; asmaa.abdelkereim@fue.edu.eg; 3 Institute of Global Health and Human Ecology, School of Sciences & Engineering, The American University in Cairo, AUC Avenue, P.O. Box 74, New Cairo 11835, Egypt; soad.nasr@aucegypt.edu (S.N.); anwar.abdelnaser@aucegypt.edu (A.A.); 4 Department of Pharmaceutics and Biopharmaceutics, University of Marburg, Robert-Koch-Str. 4, 35037 Marburg, Germany

In the original publication [1], there was a mistake in Figure 1 as published. Figure 1E (OX/PEG-CS NPs) was unintentionally assembled using a magnified image of AA-OX/PEG-CS NPs (Figure 1F). This duplication occurred during figure assembly, when magnified images from the same TEM session were organized, and panel E was mistakenly replaced. The corrected Figure 1 appears below. The authors state that the scientific conclusions are unaffected. This correction was approved by the Academic Editor. The original publication has also been updated.

## Figures and Tables

**Figure 1:**
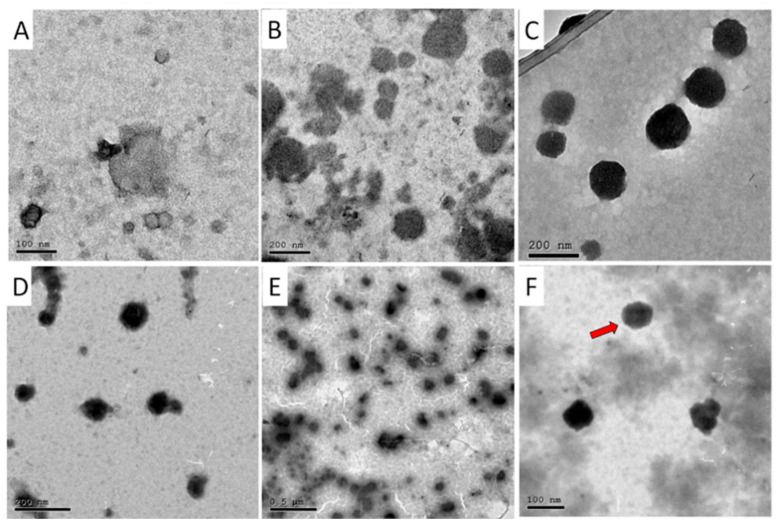
TEM images for (**A**) AA/CS NPs, (**B**) OX/CS NPs, (**C**) AA-OX/CS NPs, (**D**) AA/PEG-CS NPs, (**E**) OX/PEG-CS NPs, and (**F**) AA-OX/PEG-CS NPs. The arrow shows the PEG layer coating the CS NPs.

## References

[1] Fahmy S.A., Ramzy A., Mandour A.A., Nasr S., Abdelnaser A., Bakowsky U., Azzazy H.M.E.-S. (2022). PEGylated Chitosan Nanoparticles Encapsulating Ascorbic Acid and Oxaliplatin Exhibit Dramatic Apoptotic Effects against Breast Cancer Cells. Pharmaceutics.

