# Supplementary material for: PEGylated Chitosan Nanoparticles Encapsulating Ascorbic Acid and Oxaliplatin Exhibit Dramatic Apoptotic Effects against Breast Cancer Cells
Source: Pharmaceutics. 2022 Feb 13;14(2):407. doi: 10.3390/pharmaceutics14020407 (PMC8874531; doi:10.3390/pharmaceutics14020407)
Supplement: Supplementary file 1 [file pharmaceutics-14-00407-s001.zip › pharmaceutics-1596632-supplementary.pdf]

pharmaceutics

Supplementary Materials: PEGylated Chitosan Nanoparticles Encapsulating Ascorbic Acid and Oxaliplatin Exhibit Dramatic Apoptotic Effects against Breast Cancer Cells

Sherif Ashraf Fahmy, Asmaa Ramzy, Asmaa A. Mandour, Soad Nasr, Anwar Abdelnaser, Udo Bakowsky and Hassan Mohamed El-Said Azzazy

1. Method Validation

1.1. Linearity

Linear relationships were obtained between the peak areas and the corresponding concentrations of each component in the range of 5.00 – 100.00 µg/ mL for both VIT C and OXA. A calibration curve was constructed for each drug by plotting concentration (C) against the peak area (PA) as in Figure S1. The regression equations were computed eq 1 and 2 and regression coefficient was determined. LOD and LOQ was 1.7 and 5 µg/mL for OXA.

$PA_{VIT\ C}$ = 80.624x + 17.79; R^2 = 0.9985, (1)

PA_{OXA} = 7.2214x + 3.1358, R^2 = 0.9998, (2)

Where (PA) is the peak area, (C) is the concentration in µg/mL and (R^2) is the regression coefficient.

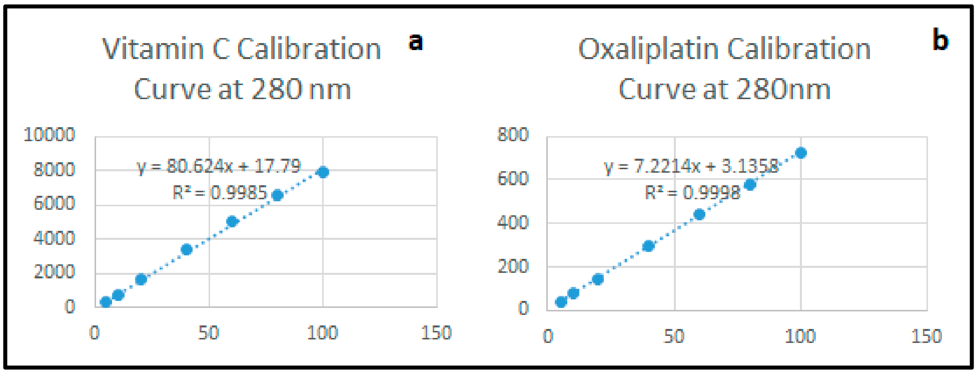

Figure S1. Calibration curves of (**a**) AA, (**b**) OX over the concentration range of 5-100 µg/mL.

1.2. Selectivity/Specificity

Specificity was tested by achieving complete baseline separation between the 2 analytes as shown in Figure S2 with good resolution compared to individual stock solution measurement.

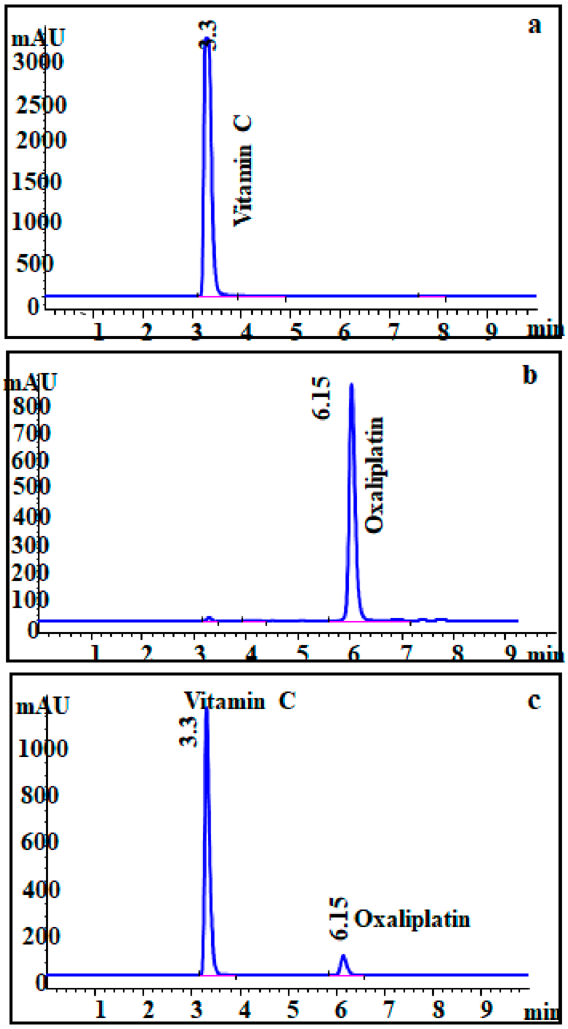

Figure S2. UHPLC chromatogram of 1µL injection of (**a**) AA (500µg/mL), (**b**) OX (1000µg/mL) and (**c**) Lab prepared mixture of AA and OX (100µg/mL).

1.3. Accuracy

The accuracy of the investigated method was validated by analyzing pure samples of both AA and OX with concentrations of 20, 40, 60 µg/mL with good results of Mean ±S.D. equals 102.56 ±1.20 & 100.35 ±1.18 for AA and OX, respectively.
